# Radiation-Induced Cellular Senescence Reduces Susceptibility of Glioblastoma Cells to Oncolytic Vaccinia Virus

**DOI:** 10.3390/cancers15133341

**Published:** 2023-06-25

**Authors:** Quinn T. Storozynsky, Xuefei Han, Shae Komant, Kate C. Agopsowicz, Kyle G. Potts, Armin M. Gamper, Roseline Godbout, David H. Evans, Mary M. Hitt

**Affiliations:** 1 Department of Oncology, University of Alberta, Edmonton, AB T6G 1Z2, Canada; 2 Li Ka Shing Institute of Virology, University of Alberta, Edmonton, AB T6G 2E1, Canada; 3 Cancer Research Institute of Northern Alberta (CRINA), University of Alberta, Edmonton, AB T6G 2R3, Canada; 4 Department of Medical Microbiology and Immunology, University of Alberta, Edmonton, AB T6G 2R3, Canada; 5 Alberta Children’s Hospital Research Institute, Faculty of Medicine, University of Calgary, Calgary, AB T2N 4N1, Canada; 6 Arnie Charbonneau Cancer Institute, Faculty of Medicine, University of Calgary, Calgary, AB T2N 4Z6, Canada; 7 Alberta Cellular Therapy and Immune Oncology (ACTION) Initiative, Faculty of Medicine, University of Calgary, Calgary, AB T2N 4Z6, Canada; 8 Cross Cancer Institute, Edmonton, AB T6G 1Z2, Canada

**Keywords:** radiation, senescence, oncolytic viruses, vaccinia virus, glioblastoma

## Abstract

**Simple Summary:**

Novel therapeutic approaches combining oncolytic virus (OV) therapy and radiotherapy are being investigated to improve treatment for glioblastoma (GBM). However, following radiotherapy cellular senescence is induced in a portion of GBM cells and it is unknown how the radiation-induced senescent state may impact the therapeutic potential of OVs. The aim of this study was to evaluate oncolytic properties of a vaccinia virus (VACV) mutant, ∆*F4L*Δ*J2R*, as well as wild-type VACV in radiation-induced senescent GBM cells. We find that both viruses exhibited attenuated phenotypes towards GBM cells under irradiated senescence-enriched conditions relative to non-irradiated controls, suggesting senescence-associated antiviral activity and underscoring the important considerations for treatment strategies combining VACV-based OVs with radiotherapy.

**Abstract:**

Glioblastoma (GBM) is a malignant brain cancer refractory to the current standard of care, prompting an extensive search for novel strategies to improve outcomes. One approach under investigation is oncolytic virus (OV) therapy in combination with radiotherapy. In addition to the direct cytocidal effects of radiotherapy, radiation induces cellular senescence in GBM cells. Senescent cells cease proliferation but remain viable and are implicated in promoting tumor progression. The interaction of viruses with senescent cells is nuanced; some viruses exploit the senescent state to their benefit, while others are hampered, indicating senescence-associated antiviral activity. It is unknown how radiation-induced cellular senescence may impact the oncolytic properties of OVs based on the vaccinia virus (VACV) that are used in combination with radiotherapy. To better understand this, we induced cellular senescence by treating GBM cells with radiation, and then evaluated the growth kinetics, infectivity, and cytotoxicity of an oncolytic VACV, ∆*F4L*Δ*J2R*, as well as wild-type VACV in irradiated senescence-enriched and non-irradiated human GBM cell lines. Our results show that both viruses display attenuated oncolytic activities in irradiated senescence-enriched GBM cell populations compared to non-irradiated controls. These findings indicate that radiation-induced cellular senescence is associated with antiviral activity and highlight important considerations for the combination of VACV-based oncolytic therapies with senescence-inducing agents such as radiotherapy.

## 1. Introduction

Glioblastoma, previously called glioblastoma multiforme (GBM), is a malignant and incurable brain cancer with a dismal prognosis despite an aggressive standard of care involving surgery, radiotherapy, and temozolomide (TMZ) administration [1]—thus, the development of novel strategies is required to treat this disease. Oncolytic virus (OV) therapy is one such developing anticancer strategy being explored clinically to improve GBM outcomes [2]. OVs infect and spread throughout tumors, eliminating cancer cells both directly via oncolysis, and indirectly through the activation of anticancer immune responses [3]. Despite its promise for GBM treatment in the clinic, OV therapy as a single modality is unable to achieve complete disease clearance [2], and therefore is increasingly being explored in combination with other established anticancer modalities [4].

Many approved therapies induce cellular senescence in cancer cells [5]. Cellular senescence is a state in which cells cease to proliferate but remain viable and metabolically active [6]. Several features that characterize senescent cells in vitro include an altered morphology (enlarged size), senescence-associated-β-galactosidase (SA-β-gal) activity, upregulation of negative regulators of the cell-cycle, such as p21^CIP1^ and p16^INK4a^, and the senescence-associated secretory phenotype (SASP; a phenotype in which senescent cells secrete a variety of defined factors that can have pro-tumorigenic downstream effects) [6,7]. Both TMZ [8] and radiation [9]—staple parts of GBM standard of care—as well as GBM salvage therapies such as lomustine [10], can induce the cellular senescence of GBM cells. Further, radiation is often investigated in combination with OV therapy to improve therapeutic outcomes [11]; therefore, we focused our investigations on radiation-induced senescence. Previous studies have demonstrated that the interaction of viruses with senescent cells is nuanced and complex [12]. Some lines of evidence suggest that cellular senescence can benefit or be exploited by viruses [13,14], whereas other reports indicate that cellular senescence is associated with antiviral activity [15,16,17]. Thus, we were interested in evaluating how OV activity may be impacted by the radiation-induced senescence of GBM cells.

Several oncolytic agents are based on vaccinia virus (VACV), a large DNA virus used to vaccinate against smallpox [18]. VACV-based oncolytic therapies have been investigated in human clinical trials [19,20] and have demonstrated efficacy in treating a variety of preclinical cancer models reviewed in [21], including several GBM models [22,23,24]. Further, radiation combined with VACV-based oncolytic therapies has been studied in a broad range of preclinical cancer models [25,26,27,28,29,30], and several preclinical GBM models [31,32,33,34]; however, VACV interaction with radiation-induced senescent cancer cells has not been evaluated.

The OV VACV used here, Δ*F4L*Δ*J2R*, is rendered dependent on host cell production of dNTPS via two viral nucleotide biosynthesis gene deletions, *F4L* (i.e., viral R2—the small subunit of viral ribonucleotide reductase), and *J2R* (i.e., viral thymidine kinase [TK]) [35]. This oncolytic Δ*F4L*Δ*J2R* VACV previously demonstrated anticancer efficacy in treating bladder cancer models [36] and an aggressive GBM model when used in combination with radiotherapy [34].

Using oncolytic Δ*F4L*Δ*J2R* VACV, as well as wild-type (WT) VACV for comparison, we investigated the growth kinetics, infectivity, and cytotoxicity of these viruses following the infection of radiation-induced senescent GBM cell populations and non-irradiated controls in vitro. We show that both oncolytic Δ*F4L*Δ*J2R* VACV and WT VACV display attenuated phenotypes in irradiated senescence-enriched GBM cell populations—indicating that, at least in vitro, radiation-induced cellular senescence impairs VACV. These findings underscore important considerations for the combination of VACV-based oncolytic therapies with senescence-inducing agents and provide evidence that cellular senescence induces antiviral properties.

## 2. Materials and Methods

### 2.1. Cell Lines and Culture Conditions

Human GBM cell lines U87 (obtained from Jorgen Fogh, Sloane Kettering Institute, Rye, NY, USA), U118 (American Type Cell Culture Collection (ATCC); HTB-15), and T98 (obtained from Walter Nelson-Rees, Naval Biomedical Research Station, Oakland, CA, USA) were maintained in high glucose Dulbecco’s modified Eagle’s medium (HG-DMEM) supplemented with 5% fetal bovine serum (FBS), 2 mM L-glutamine, 100 U/mL penicillin, and 100 μg/mL streptomycin (Gibco, Billings, MT, USA). The African green monkey kidney (AGMK) cell line BSC-40 (CRL-2761) was maintained in a minimal essential medium (MEM) supplemented as above. Vero (CCL-81), another AGMK cell line, was cultured in HG-DMEM supplemented with 10% FBS, plus glutamine, penicillin, and streptomycin as above. L929 mouse fibroblast cells (kindly provided by M. Shmulevitz, University of Alberta) were maintained in MEM supplemented with 1× nonessential amino acids (Millipore Sigma, Burlington, MA, USA) and 1 mM sodium pyruvate (Millipore Sigma). The patient-derived GBM cell line ED501 (established by Hua Chen [37], University of Alberta) was maintained in serum-free DMEM/F12 medium supplemented with 20 ng/mL recombinant human epidermal growth factor (Gibco), 10 ng/mL human basic fibroblast growth factor (Cedarlane, Burlington, Canada), 0.2% heparin (Sigma-Aldrich, St. Louis, MO, USA), and 1× B-27^®^ Supplement (Gibco). The consent for patient GBM tissue was obtained prior to surgery under the Health Research Ethics Board of Alberta Cancer Committee Protocol #HREBA CC-14-0070. The ED501 cell line was passaged using Accutase (Gibco); all other cell lines were passaged using trypsin (Gibco). Cells were cultured at 37 °C in a humidified 5% CO_2_ environment. Cells were tested for mycoplasma contamination using either DAPI (Sigma-Aldrich) staining and fluorescence microscopy [38], or a Mycoplasma PCR Detection Kit (Applied Biological Materials, Richmond, Canada).

### 2.2. Viruses

The WT VACV Western Reserve strain was initially obtained from the ATCC. Homologous recombination techniques were used to construct the oncolytic Δ*F4L*Δ*J2R* VACV from a clonal isolate of WT VACV as described elsewhere [36]. VACV stocks were produced as previously described [39]. Serotype 3 Dearing (T3DPL) reovirus kindly provided by M. Shmulevitz (University of Alberta, Edmonton, AB, Canada) was originally from P. Lee (Dalhousie University, Halifax, Nova Scotia, Canada). Reovirus stocks were produced as previously described [40]. VSVΔM51-GFP (Indiana strain) was engineered as described by Stojdl et al. (2003) [41] and was kindly provided by Doug Mahoney (University of Calgary, Calgary, AB, Canada). VSVΔM51-GFP was propagated in monolayer cultures of Vero cells and purified using standard protocols [42].

### 2.3. In Vitro Irradiation and Induction of Senescence-Enriched Cell Cultures

GBM cells, including parallel cultures for later use in cell counting, were treated with a radiation dose of 10 Gy using a MultiRad 160 X-ray irradiator (Faxitron, Tucson, AZ, USA) or non-irradiated (0 Gy control cells). Cells were then cultured in a growth medium appropriate for the cell line in a humidified 5% CO_2_ environment at 37 °C for 7 days.

### 2.4. Cell Proliferation Assays

For proliferation assays, duplicate wells in 6-well plates were seeded with 1 × 10^4^ cells. Then 24 h later, GBM cells were either irradiated or not irradiated. At the indicated time points post-irradiation, the cells were washed with 1× phosphate buffered saline (PBS), detached by trypsinization, and diluted 1:1 in trypan blue (Lonza, Rockland, ME, USA). The total cells per well were counted using a hemacytometer (Hausser Scientific, Horsham, PA, USA). For assays to assess the proliferation of serum-starved cells, duplicate wells in 12-well plates were seeded with 4 × 10^4^ cells into medium containing either 0.1% FBS or 5% FBS. At the indicated time points post-seeding, the total cells per well were calculated as described above.

### 2.5. Reverse Transcription Quantitative Polymerase Chain Reaction (RT-qPCR)

Total RNA was isolated from non-irradiated and irradiated GBM cells 7 days post-irradiation using the illustra™ RNAspin Mini Kit (GE Healthcare, Chicago, IL, USA) according to the manufacturer’s instructions. Isolated RNA (2 µg) was used as a template to synthesize complementary DNA (cDNA) using the High-Capacity cDNA Reverse Transcription Kit (Applied Biosystems, Waltham, MA, USA) according to the manufacturer’s instructions. RT-qPCR analysis was performed using diluted cDNA (1:30) and Fast SYBR™ Green Master Mix (Applied Biosystems) in Optical 96-Well Fast Thermal Cycling Plates (Applied Biosystems). Target gene expression levels were normalized to 18S rRNA. The primer sequences are listed in Table S1.

### 2.6. Senescence-Associated β-Galactosidase Activity

Non-irradiated and irradiated GBM cells were assessed for SA-β-gal activity 7 days post-irradiation using the Senescence Beta-Galactosidase Staining Kit (Cell Signaling) according to the manufacturer’s instructions. Briefly, the cells were washed with 1× PBS, incubated at room temperature for 15 min in fixative solution, washed twice with 1× PBS, then incubated at 37 °C in a dry incubator (no CO_2_) in a β-galactosidase staining solution for 12–30 h as needed for sufficient color development. Next, the cells were washed twice with 1× PBS, overlaid with 70% glycerol, and imaged using an Olympus IX70 microscope (Olympus Life Sciences, Center Valley, PA, USA). SA–β-gal positive (blue-colored) cells were quantified manually using ImageJ software (Version 1.51w) to view the images.

### 2.7. Immunoblotting

Whole cell lysates were prepared from non-irradiated and irradiated GBM cells 7 days post-irradiation using RIPA lysis buffer (150 mM sodium chloride, 1% Triton X-100, BioRad, 0.5% sodium deoxycholate, 0.1% sodium dodecyl sulfate, 50 mM Tris-HCl (pH 8.0), 0.1 mg/mL phenylmethylsulfonyl fluoride, 1× Halt™ protease inhibitor cocktail, Thermo Fisher, Waltham, MA, USA). The protein concentration was quantified using the Pierce™ BCA Protein Assay Kit (Thermo Fisher) according to the manufacturer’s instructions. Next, the protein was resolved by SDS-PAGE and transferred to Immobilon-FL polyvinylidene difluoride (PVDF) membranes (EMD Millipore, Burlington, MA, USA). The PVDF membranes were blocked using Odyssey blocking buffer (Li-COR Biosciences, Lincoln, NE, USA), washed, then incubated overnight at 4 °C with the following primary antibodies diluted at the indicated concentrations in blocking buffer containing 0.2% Tween 20 (Fisher Scientific): anti-p21 (1:1000; ab109520, Abcam, Cambridge, UK), anti-TK1 (1:500; ab59271, Abcam), anti-RRM2 (1:500; ab57653, Abcam), anti-p53R2 (1:1000; ab8105 [Abcam]), anti-β-tubulin (1:1000; 2146, Cell Signaling Technologies, Danvers, MA, USA), or anti-β-actin (1:1000; 926-42210, Li-COR Biosciences). Next, the PVDF membranes were washed and then incubated at room temperature for 1 h with the following fluorescent-dye conjugated secondary antibodies diluted at the indicated concentrations in blocking buffer containing 0.2% Tween 20 (Fisher Scientific) and 0.01% sodium dodecyl sulfate: IRDye^®^ 680RD Goat anti-Mouse (1:20,000; 926-68,070, Li-COR Biosciences) or IRDye^®^ 800CW Donkey anti-Rabbit (1:20,000; 926-32,213, Li-COR Biosciences). The PVDF membranes were washed and then scanned using an Odyssey Infrared Imaging System (Li-COR Biosciences) and analyzed using Image Studio version 2.0 software (Li-COR Biosciences).

### 2.8. Virus Growth Curves

Non-irradiated and irradiated GBM cells were cultured for 7 days to allow the induction of senescence. Using cultures prepared in parallel to experimental cultures, the cells were counted using trypan blue as described in Section 2.4 to confirm viability, to ensure that equal numbers of cells had been plated in 12-well plates, and for the multiplicity of infection (MOI) calculations. The cells were then infected with the virus at an MOI of 0.03 plaque-forming units (PFU) or 3.0 PFU per cell, as indicated, in 12-well plates. Lysates were collected at the indicated times, frozen, and thawed three times, then titered in duplicate on BSC-40 cells as previously described [36], except without carboxymethylcellulose. The reovirus growth experiments were performed similarly and titered on L929 cells as previously described [40]. VSVΔM51-GFP growth experiments were performed similarly and titered as described for VACV, except using Vero cells overlaid with DMEM supplemented with 5% FBS and 1% carboxymethyl cellulose.

### 2.9. Quantification of Virus Infection and Cellular Senescence

The retention of the fluorescent dye, CellTrace™ Violet (CTV), was used as a surrogate marker for cellular senescence. The cells were stained using the fluorescent CellTrace™ Violet Cell Proliferation Kit (Invitrogen, Waltham, MA, USA) according to the manufacturer’s instructions. Mock-stained cells (with or without VACV-infection) were used as controls for the background CTV fluorescence. Briefly, a single-cell suspension of 1 × 10^6^ cells in 0.5 mL of 5–10 µM CTV staining solution was incubated for 20 min at 37 °C. A volume of culture medium (HG-DMEM supplemented as described above) that was 5–6 times the staining solution volume was added, and the cell solution was incubated for 5 min at room temperature to quench the free CTV. The cells were pelleted, resuspended in cell line specific culture medium, and treated with the indicated radiation doses (0 or 10 Gy). Then 7 days post-irradiation, cells in duplicate wells of black 96-well plates (Greiner, Kremsmünster, Austria) were infected with the indicated VACVs at 3.0 PFU per cell. Then 24 h post-infection, the cell monolayers were analyzed for the expression of the late VACV protein, A27, as a marker for the productive infection. Mock-infected cells (with or without CTV staining) were used as controls for the background antibody fluorescence. Briefly, the cell monolayers were fixed for 20 min at room temperature using 4% paraformaldehyde, washed thrice with 1× PBS, blocked/permeabilized for 30 min using Odyssey blocking buffer (Li-COR Biosciences) containing 0.1% Triton-X100 (BioRad, Hercules, CA, USA), and then incubated for 1 h at room temperature with primary anti-A27 VACV antibody (ab117453, Abcam) diluted 1:500 in blocking buffer. The cells were washed thrice with 1× PBS and then incubated for 30 min at room temperature with fluorescent-dye conjugated secondary Goat anti-Rabbit antibody (ab150077, Abcam) diluted 1:2000 in a blocking buffer. Next, the cells were washed thrice with 1× PBS and the plates were imaged for both CTV fluorescence and anti-A27 VACV antibody fluorescence using an ImageXpress^®^ Micro High-Content System (Molecular Devices, San Jose, CA, USA). MetaXpress 6.5.5.559 software (Molecular Devices) was used to analyze and create overlays of the fluorescent images. The fluorescence intensity of images displaying non-irradiated CTV-stained cells (with or without VACV-infection) was used as a reference for low CTV intensity values; the fluorescence intensities above this reference were deemed high CTV intensity. An example of CTV-stained cells that are non-irradiated (mostly low CTV intensity—grey cells) and irradiated (mostly high CTV intensity—blue cells) is shown in Figure S1. The cells were quantified manually using GIMP 2.10.18 software to view the images. Whenever feasible, the cells were protected from light.

### 2.10. Cytotoxicity Assays

GBM cells were either irradiated or non-irradiated, then 7 days post-irradiation triplicate wells of GBM cells on 48-well plates were infected with VACV at the indicated MOIs and incubated for a total of 72 h at 37 °C. Neutral red uptake assays were performed to assess cell viability as previously outlined [43]. Briefly, the medium was replaced with culture medium containing 40 µg/mL of neutral red (Sigma-Aldrich) during the last 1–2 h of incubation. Next, the neutral red medium was removed, the cells were washed with 1× PBS, neutral red destain solution (50% ethanol, 49% deionized water, 1% glacial acetic acid) was added, and the plates were incubated/shaken for 10 min at room temperature. The fluorescence was measured using a FLUOstar plate reader (BMG Labtech, Ortenberg, Germany) with 544 nm excitation/620 nm emission filters. The cell viability was calculated from the fluorescence of the treated samples minus the background fluorescence of completely non-viable cells (treatment with 2% Triton X-100, BioRad), and is reported as a percent value relative to the fluorescence of mock infected cells (100% cell viability).

### 2.11. Conditioned Medium Experiments

GBM cells were either irradiated or non-irradiated. At 7 days post-irradiation, the medium was exchanged with fresh culture medium, and irradiated or non-irradiated cells were incubated for 48 h under normal growth parameters to condition the medium. The conditioned medium was collected, filtered using the 0.22 µm Steriflip^®^ Vacuum-driven Filtration System (Millipore, Burlington, MA, USA), and used to culture fresh radiation-naïve GBM cells for 24 h in 12-well plates. Next, the virus growth curves were generated by infecting the cells with VACVs at 0.03 PFU per cell, allowing virus growth in the presence of conditioned medium, collecting lysates at the indicated times, and titering lysates in duplicate on BSC-40 cells as above.

### 2.12. Statistics and Analysis

Data were analyzed using GraphPad Prism 9.4.1 software. To compare two groups, an unpaired *t*-test was used. Two-way analysis of variance (ANOVA) was used to compare the growth curves. A non-linear regression was used to fit curves to the cytotoxicity data and the curves were compared using extra sum-of-square F test.

## 3. Results

### 3.1. Radiation Induces Senescence in Glioblastoma Cells

The established U87 GBM cell line, as well as a patient-derived GBM cell line, ED501, were employed to assess a panel of different markers evaluating the induction of cellular senescence following ionizing radiation exposure. Cellular senescence typically takes 3–7 days to develop in GBM cells after radiation exposure, with increased numbers of radiation-induced senescent GBM cells present at late relative to early time points (5–14 days versus 1–3 days) [9,44,45,46,47]. Thus, we chose to perform our analyses 7 days post-irradiation.

**Figure 1:**
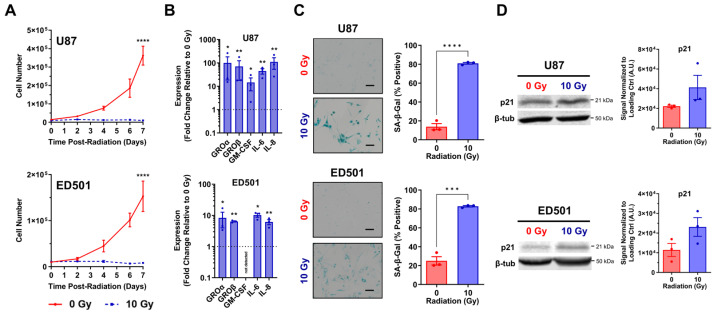
**Verification of radiation-induced senescence in irradiated glioblastoma cells.** Human U87 and ED501 glioblastoma cell lines were either non-irradiated (0 Gy) or treated with a radiation dose of 10 Gy then evaluated for the markers of cellular senescence. (**A**) Cellular growth curves showing the change in cell number over time following the indicated radiation treatments. Trypan blue assays were performed to quantify total live cells at the indicated time points. (**B**) Fold change in the expression of senescence-associated secretory phenotype (SASP) genes in 10 Gy treated cells relative to 0 Gy treated cells 7 days following radiation treatments, based on RT-qPCR analysis. The 18S rRNA levels were used for normalization. (**C**) Representative images of cells assessed for senescence-associated-β-galactosidase activity (left panels) and quantification of cells positive for senescence-associated-β-galactosidase activity (right panels) 7 days following the indicated radiation treatments. (**D**) Representative immunoblots showing p21 protein levels (left panels) and quantification of band signals (arbitrary units; A.U.) normalized to β-tubulin loading controls (right panels) 7 days following radiation treatments. **Data information:** data represent three independent experiments, mean ± SEM is shown. For (**A**), significance was determined by two-way ANOVA. For (**B**), asterisks indicate significance of unpaired *t*-test comparing ΔCt values (Figure S3). For (**C**), unpaired *t*-test was used to determine significance and scale bar = 100 µm. (* = *p* < 0.05; ** = *p* < 0.01; *** = *p* < 0.001; **** = *p* < 0.0001). For (**D**), the uncropped immunoblots are shown in Supplementary Material File S1.

Cell proliferation ceased in irradiated U87 and ED501 cell lines while non-irradiated controls continued to proliferate over a 7-day period based on trypan blue assays (Figure 1A). Additionally, 7 days after radiation treatments, the transcription of SASP-associated genes was significantly increased in irradiated GBM cells compared to non-irradiated controls (Figure 1B). At this same 7-day time point, irradiated GBM cells were larger in size (Figure 1C; left panels) and had significantly increased SA–β-gal activity compared to non-irradiated controls, with the vast majority of irradiated U87 and ED501 cells staining positive for SA–β-gal activity (~80%) (Figure 1C; right panels). The same trends were observed using two additional human GBM cells lines, T98 and U118 (Figure S2). There was a trend towards increased levels of p21, a negative regulator of the cell-cycle, in irradiated U87 and ED501 cells relative to non-irradiated controls 7 days after treatments (Figure 1D), and no increase was observed in irradiated T98 and U118 cells (Figure S2D). Collectively, these data indicate that radiation-induced senescent GBM cells are reliably generated 7 days following ionizing radiation exposure.

### 3.2. Vaccinia Virus Growth Is Attenuated in Irradiated Senescence-Enriched Glioblastoma Cell Populations

**Figure 2:**
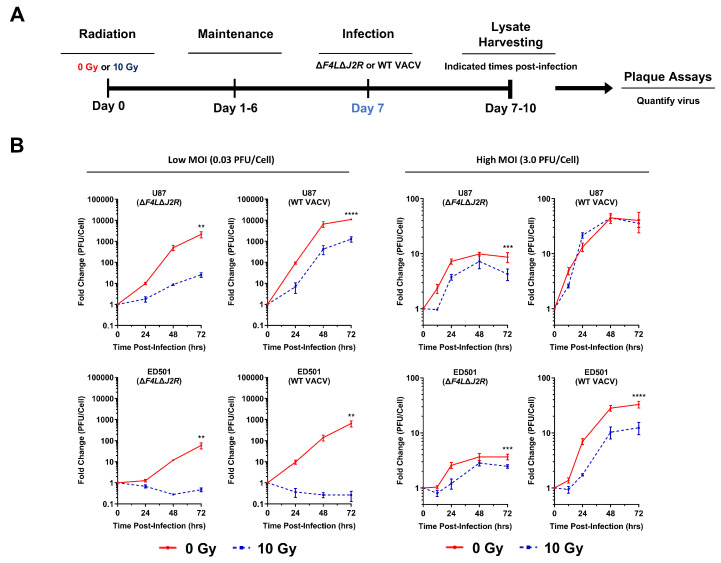
**Vaccinia virus replication is attenuated in irradiated senescence-enriched human glioblastoma cell lines.** (**A**) Experimental outline: human U87 and ED501 glioblastoma cell lines were either non-irradiated (0 Gy) or treated with a radiation dose of 10 Gy. Then, 7 days later, cells were infected with the indicated vaccinia viruses. Lysates were harvested at the indicated times and titered by plaque assay to assess virus yield. (**B**) Amplification of oncolytic ∆*F4L*∆*J2R* and wild-type (WT) vaccinia viruses in 0 Gy (non-irradiated; solid red lines) and 10 Gy (dashed blue lines) treated human U87 and ED501 glioblastoma cell lines. Cells were infected with 0.03 PFU per cell (left panels) or 3.0 PFU per cell (right panels). **Data information:** data represent three independent lysates titered in duplicate, mean ± SEM is shown. Graphs show fold change relative to lysates taken at t = 0. Significance determined by two-way ANOVA analysis (** = *p* < 0.01; *** = *p* < 0.001; **** = *p* < 0.0001).

We next investigated the growth kinetics of oncolytic Δ*F4L*Δ*J2R* VACV and WT VACV in U87 and ED501 cell lines under the same treatment conditions as above. Virus growth was assessed as outlined in Figure 2A. Briefly, GBM cells were irradiated to induce cellular senescence (or not), then infected 7 days later with virus at a low MOI (0.03 PFU per cell) and the virus yields were determined over a 72-h growth period. The virus growth curves indicated that the amplification of oncolytic Δ*F4L*Δ*J2R* VACV and WT VACV was significantly reduced in irradiated senescence-enriched U87 and ED501 cell lines compared to non-irradiated controls (Figure 2B; left panels). This effect was most pronounced with the patient-derived ED501 cell line, with no virus growth observed under the irradiated senescence-enriched conditions (Figure 2B; left panels). Additionally, attenuated growth of oncolytic Δ*F4L*Δ*J2R* VACV and WT VACV was observed using T98 and U118 GBM cell lines treated the same way (Figure S4A,B). We extended our analysis of virus growth kinetics by treating GBM cells as above, except using a 100-fold higher MOI (3.0 PFU per cell) to ensure that all cells were infected at t = 0 h. Again, oncolytic Δ*F4L*Δ*J2R* VACV was attenuated in both irradiated senescence-enriched U87 and ED501 cell lines versus non-irradiated controls; however, WT VACV was only attenuated in the ED501 cell line, not the U87 cell line (Figure 2B; right panels). These data suggest that at a high MOI, the attenuation of WT VACV growth is cell-line dependent.

Next, we cultured U87 cells under low-serum conditions to restrict cellular proliferation. Serum-starved U87 cells did not proliferate over 72 h but did under normal-serum conditions (Figure S5A). Using low-serum culture conditions, we assessed the growth of oncolytic Δ*F4L*Δ*J2R* VACV and WT VACV in irradiated and non-irradiated U87 cells infected 7 days post-irradiation with a low MOI (0.03 PFU per cell). Again, the growth of oncolytic Δ*F4L*Δ*J2R* VACV was significantly attenuated in the irradiated senescence-enriched condition compared to non-irradiated (non-proliferating) controls (Figure S5). The growth of WT VACV was also reduced by ~45% 72 h post-infection in the irradiated senescence-enriched condition versus non-irradiated (non-proliferating) controls, although non-significantly (Figure S5). These data indicate that the decreased VACV growth observed in irradiated senescence-enriched conditions compared to non-irradiated controls is not solely due to the increased proliferation of non-irradiated control cells.

Lastly, to better understand whether this attenuated virus growth was specific to VACV or applicable to other viruses used for OV therapy, we performed additional experiments using a reovirus and the vesicular stomatitis virus (VSV) mutant, VSVΔM51-GFP. The reovirus growth was also attenuated in irradiated senescence-enriched U87 cells relative to non-irradiated controls (Figure S6). Of note, the growth of the reovirus was blocked in both irradiated and non-irradiated patient-derived ED501 cells, suggesting that the pathways other than those implicated in senescence must restrict the reovirus infection of these cells (Figure S6). Interestingly, unlike VACV, the growth of VSVΔM51-GFP was unchanged between the irradiated senescence-enriched ED501 cells and non-irradiated controls in both low MOI (0.03 PFU per cell) and high MOI (3.0 PFU per cell) conditions (Figure S7). VSVΔM51-GFP growth was also unchanged between irradiated senescence-enriched cells and non-irradiated controls with the U87 cell line infected using a high MOI (3.0 PFU per cell); however, using a low MOI (0.03 PFU per cell), the growth of VSVΔM51-GFP was slightly, but statistically, attenuated in the irradiated senescence-enriched condition (Figure S7).

Overall, these data indicate that VACV growth is attenuated in irradiated senescence-enriched GBM cell lines. Further, the virus growth attenuation in irradiated senescence-enriched conditions is virus dependent.

### 3.3. Infectivity of Vaccinia Virus Is Reduced in Irradiated Senescence-Enriched Glioblastoma Cell Populations

**Figure 3:**
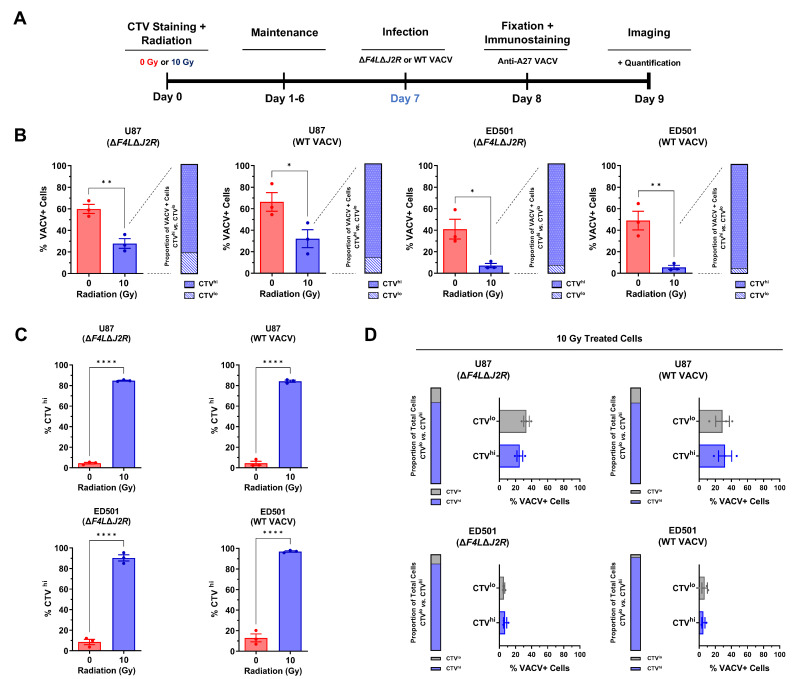
**Vaccinia virus infectivity is reduced in irradiated senescence-enriched human glioblastoma cell lines.** (**A**) Experimental outline: human U87 and ED501 glioblastoma cell lines were stained with a fluorescent cell proliferation marker (CellTrace™ Violet; CTV) then were either non-irradiated (0 Gy) or treated with a radiation dose of 10 Gy. Then 7 days later, cells were infected with 3.0 PFU per cell of oncolytic Δ*F4L*∆*J2R* or wild-type (WT) vaccinia viruses. At 24 h post-infection, cells were fixed then immunostained with an antibody against the late A27 VACV protein and imaged the next day using fluorescence microscopy. (**B**) Left panels: percent VACV+ cells 24 h post-infection of 0 Gy or 10 Gy treated cells (note: all cells were also treated with CTV). Right panels: proportion of VACV+ irradiated cells that were CTV^hi^ vs. those that were CTV^lo^. (**C**) Percent of cells scored as high CTV fluorescence (CTV^hi^, indicating few or no cell divisions) 8 days following CTV staining and treatment with 0 Gy or 10 Gy (note: all cells were treated with virus as indicated). (**D**) Left panels: proportion of irradiated cells that were CTV^hi^ vs. those that were CTV^lo^ 8 days following CTV staining (note: all cells were treated with virus as indicated). Right panels: percentage of irradiated cells that were VACV+ in the CTV^hi^ population and in the CTV^lo^ population from the same cultures. **Data information:** data represent three independent experiments, mean ± SEM is shown for (B—left panels), (C), and (D—right panels). For (B—right panels, and D—left panels), the mean value from three independent experiments is shown. For (B—left panels), (C), and (D—right panels), significance determined using unpaired *t*-test (* = *p* < 0.05; ** = *p* < 0.01; **** = *p* < 0.0001).

To better understand the attenuated phenotype of VACV in irradiated senescence-enriched cells, we stained U87 and ED501 cells with a fluorescent proliferation marker (CellTrace™ Violet; CTV) to label radiation-induced senescent cells. Non-proliferating cells retain high fluorescence intensities whereas proliferating cells lose fluorescence intensity at each division. As outlined in Figure 3A, 7 days following radiation treatments and CTV staining, we infected GBM cells with oncolytic ∆*F4L*∆*J2R* VACV or WT VACV and evaluated the number of VACV+ infected cells by staining with an antibody against the late A27 VACV protein 24 h post-infection.

In comparison to non-irradiated controls, the percentage of VACV+ cells 24 h post-infection with oncolytic ∆*F4L*∆*J2R* VACV and WT VACV was significantly reduced in irradiated senescence-enriched conditions (Figure 3B; left panels). These data indicate that VACV is less infectious in GBM cell populations that are enriched with senescent cells post-irradiation. To better understand the interaction of VACV with radiation-induced senescent cells, we analyzed 10 Gy-VACV+ cells to determine whether these cells displayed a high CTV intensity (CTV^hi^) or a low CTV intensity (CTV^lo^). The majority (~85–95%) of 10 Gy-VACV+ cells were CTV^hi^ (Figure 3B; right panels), suggesting that VACV is indeed capable of productively infecting radiation-induced senescent cells, as opposed to being restricted to irradiated CTV^lo^ cells.

In agreement with our senescence marker analysis in Figure 1, the majority (~80–95%) of irradiated GBM cells were CTV^hi^ compared to non-irradiated controls (Figure 3C), indicating enrichment for senescent cells post-irradiation, but also a sparse population of CTV^lo^ cells (~5–20%; Figure 3D; left panels). We analyzed the population of virus-treated irradiated cells to determine the percentage of VACV+ cells within CTV^hi^ and CTV^lo^ categories. Interestingly, there was no significant difference between the percentage of VACV+ cells within the CTV^hi^ category and that in the CTV^lo^ category (Figure 3D; right panels). Put another way, following a 10 Gy dose of radiation, both radiation-induced senescent GBM cells (CTV^hi^) and a sparse population of proliferating GBM cells (CTV^lo^) are equally likely to be infected, albeit both at lower percentages than non-irradiated controls.

All together, these data indicate that in the irradiated senescence-enriched condition, both the radiation-induced senescent GBM cells and a sparse proliferating population of GBM cells are resistant to VACV infection relative to non-irradiated controls.

### 3.4. Reduced Vaccinia Virus Cytotoxicity in Irradiated Senescence-Enriched Glioblastoma Cell Populations

**Figure 4:**
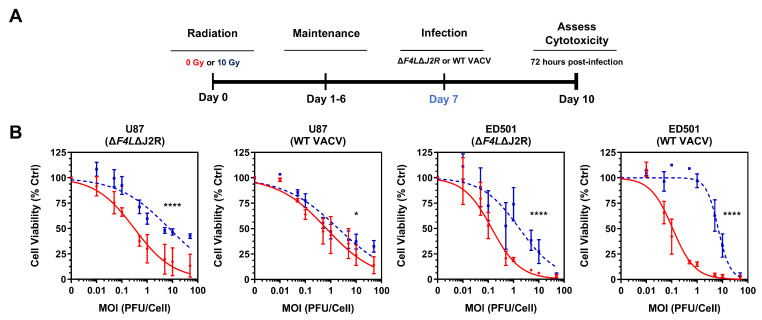
**Vaccinia virus is less cytotoxic to irradiated senescence-enriched human glioblastoma cell lines than to non-irradiated cell lines.** (**A**) Experimental outline: human U87 and ED501 glioblastoma cell lines were either non-irradiated (0 Gy) or treated with a radiation dose of 10 Gy. Then 7 days later, cells were infected with the indicated vaccinia viruses. At 72 h post-infection, neutral red cell viability assays were performed to assess cytotoxicity. (**B**) Dose-response curves showing cell viability 72 h post-infection with the indicated vaccinia viruses in 0 Gy (non-irradiated; solid red lines) and 10 Gy (dashed blue lines) treated glioblastoma cell lines. **Data information**: data represent three independent experiments. Mean ± SEM is shown for data points. For (**B**), data are normalized to mock-infected control (0 PFU per cell = 100% cell viability) and curves fit to data points using nonlinear regression and analyzed by extra sum-of-square F test (* = *p* < 0.05; **** = *p* < 0.0001).

We next evaluated the cytotoxicity of oncolytic ∆*F4L*∆*J2R* VACV and WT VACV towards irradiated senescence-enriched U87 and ED501 cell lines using the neutral red viability assay. As depicted in Figure 4A, the GBM cells were irradiated to induced cellular senescence (or left untreated), then infected 7 days later with increasing concentrations of virus and assessed for cell viability 72 h post-infection.

The dose-response curves evaluating the cell viability indicated that both oncolytic ∆*F4L*∆*J2R* VACV and WT VACV were significantly less cytotoxic towards irradiated senescence-enriched U87 and ED501 cell lines compared to the non-irradiated controls (Figure 4B). These data indicate that irradiated senescence-enriched GBM cell populations are less susceptible to VACV-mediated cell killing, consistent with our data on virus growth in these cells.

### 3.5. Oncolytic ∆F4L∆J2R Vaccinia Virus Attenuation Is Not Explained by Reduced Cellular Nucleotide Biosynthesis Machinery

VACV requires the production of dNTPs to construct new virus genomes during the viral replication process. To this end, VACV triggers host cells to accumulate in the S-phase [48,49], a point in the cell-cycle in which the cellular proteins involved in nucleotide biosynthesis are upregulated, such as cellular TK and cellular ribonucleotide reductase small subunit R2 (RRM2) [50,51,52]. Additionally, VACV encodes a plethora of its own viral nucleotide biosynthesis machinery to ensure dNTP production [53].

A possible explanation for the attenuated phenotype of oncolytic ∆*F4L*∆*J2R* VACV in irradiated cells is that irradiated cells are deficient in the cellular nucleotide biosynthesis enzymes necessary to compensate for the deleted viral genes *J2R* (viral TK) and *F4L* (viral R2). Previous studies have demonstrated that the downregulation of cellular TK reduces *J2R*-deleted VACV growth [54], and the downregulation of cellular R2 reduces *F4L*-deleted VACV and ∆*F4L*∆*J2R* VACV growth (but not *J2R*-deleted growth) [36]. Furthermore, cellular p53R2 is an R2-related enzyme that is upregulated following radiation exposure [55]. Thus, to better understand the attenuated phenotype of ∆*F4L*∆*J2R* VACV, we used immunoblotting to evaluate the protein levels of cellular TK1, RRM2, and p53R2 in irradiated senescence-enriched GBM cell lines and non-irradiated controls, expecting that a decrease in levels of these proteins might explain the attenuation of the virus in senescence cells.

**Figure 5:**
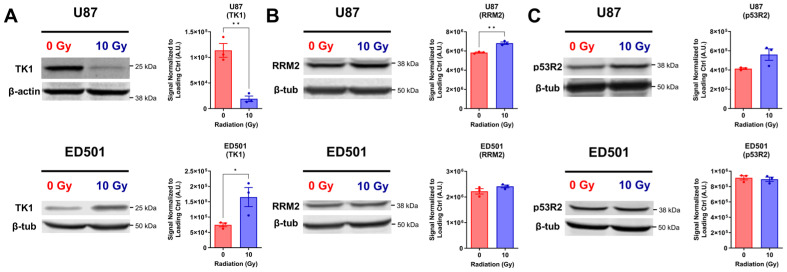
**Protein levels of cellular nucleotide biosynthesis enzymes in irradiated senescence-enriched human glioblastoma cell lines and non-irradiated controls.** Human U87 and ED501 glioblastoma cell lines were either non-irradiated (0 Gy) or treated with a radiation dose of 10 Gy. Then 7 days later, whole cellular lysates were isolated and analyzed by immunoblotting. Representative images of immunoblots (left panels) and quantified band signals (arbitrary units, A.U.; right panels) show expression of nucleotide biosynthesis enzymes: (**A**) thymidine kinase 1 (TK1); (**B**) ribonucleotide reductase regulatory subunit M2 (RRM2); (**C**) p53 inducible small subunit of ribonucleotide reductase (p53R2). β-actin or β-tubulin were used as loading controls. **Data information**: data represent three independent experiments. The quantified band signal was determined by normalizing the probed signal with a loading control signal. For (A-C; right panels), mean ± SEM is shown, and unpaired *t*-test was used to determine significance (* *p* < 0.05; ** *p* < 0.01). The uncropped immunoblots are shown in Supplementary Material File S1.

TK1 levels were significantly reduced in irradiated senescence-enriched U87 cells compared to non-irradiated controls, but significantly increased in the ED501 cell line (Figure 5A). U87 and ED501 cell lines displayed either slightly, but significantly, increased RRM2 levels (U87), a trend towards increased p53R2 levels (U87), or unchanged RRM2 and p53R2 levels (ED501) in the irradiated senescence-enriched condition compared to non-irradiated controls (Figure 5B,C). Overall, TK1 in U87 cells was the only cellular nucleotide biosynthesis enzyme downregulated by radiation treatment.

As further evidence that nucleotide biosynthesis was not a determining factor for the growth of ∆*F4L*∆*J2R* VACV in irradiated senescence-enriched conditions, we examined the growth of singly mutated VACVs under this condition. One might predict that a *J2R*-deleted VACV would display attenuated growth in irradiated senescence-enriched U87 cells and that an *F4L*-deleted VACV would not (given cellular TK1, but not cellular RRM2, downregulation following radiation treatment). However, both *J2R*-deleted VACV and *F4L*-deleted VACV displayed attenuated growth post-infection of irradiated senescence-enriched U87 cells compared to non-irradiated controls (Figure S8). Of note, *J2R*-deleted VACV, *F4L*-deleted VACV, ∆*F4L*∆*J2R* VACV, and WT VACV (Figure S8 and Figure 2B,C) all displayed growth attenuation following the radiation-induced senescence of the ED501 cell line compared to non-irradiated controls, yet no downregulation of cellular nucleotide biosynthesis enzymes following radiation treatment was observed with the ED501 cell line. In summary, decreased levels of the cellular nucleotide biosynthesis enzymes does not explain the attenuated phenotype of ∆*F4L*∆*J2R* VACV.

### 3.6. Radiation-Induced Senescence of Human Glioblastoma Cells Increases Expression of NF-κB-Associated Genes, but Not Type I Interferon Related Genes

The type I interferon (IFN) system and the NF-κB pathway are established antiviral players [56,57]. Further, radiation-induced DNA damage is implicated in activating the type I IFN system [58], and activation of the NF-κB pathway promotes cellular senescence [59,60]. Thus, we hypothesized that these antiviral-associated pathways could be active in irradiated senescence-enriched GBM cell populations and potentially responsible for VACV attenuation.

To elucidate whether these pathways were active, we assessed the expression of several genes associated with the type I IFN system (IFNβ, MX1, and ISG15) and the NF-κB pathway (IκBα, IL1β, and TNFα) in irradiated senescence-enriched GBM cell populations and non-irradiated controls 7 days following radiation treatments. IFNβ is a prototypical type I IFN that activates a plethora of IFN-stimulated genes (ISGs) [61,62]. MX1 and ISG15 are well-characterized ISGs [63,64]. IκBα is a cytoplasmic inhibitor of NF-κB that participates in a negative feedback loop to suppress NF-κB activity; its gene expression is activated by NF-κB [65]. As well, NF-κB induces the expression of genes encoding the proinflammatory factors IL1β and TNFα [66].

**Figure 6:**
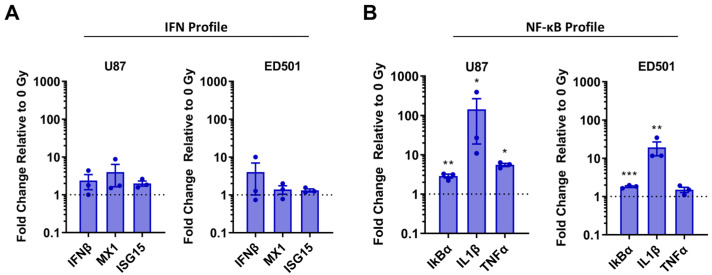
**Radiation-induced senescence of human glioblastoma cells increases expression of NF-κB-associated genes, but not type I interferon related genes.** Human U87 and ED501 glioblastoma cell lines were either non-irradiated (0 Gy) or treated with a radiation dose of 10 Gy. Then 7 days later, RNA was isolated and gene expression analyzed by RT-qPCR with 18S rRNA used for normalization. Shown is the increase in expression in 10 Gy treated relative to non-irradiated (0 Gy) glioblastoma cells of the following gene panels: (**A**) Type I interferon (IFN) related genes; (**B**) NF-κB-associated genes. **Data information**: data represent three independent experiments, mean ± SEM is shown. Asterisks indicate unpaired *t*-test comparing ΔCt values (* = *p* < 0.05; ** = *p* < 0.01; *** = *p* < 0.001; ΔCt values are shown in Figure S9).

Expression of IFNβ, MX1, and ISG15 was not significantly different between the irradiated and non-irradiated controls (Figure 6A). These data suggest that the type I IFN system is not upregulated in irradiated senescence-enriched GBM cell populations. However, the expression of IκBα and IL1β was significantly upregulated in irradiated senescence-enriched U87 and ED501 cell lines compared to non-irradiated controls (Figure 6B). Further, with the U87 cell line, the expression of TNFα was upregulated in the irradiated senescence-enriched condition compared to the non-irradiated controls (Figure 6B). Collectively, these data suggest that the NF-κB pathway may be activated in irradiated senescence-enriched GBM cell populations, but not the type I IFN system.

### 3.7. Irradiated Senescence-Enriched Human Glioblastoma Cell Populations Secrete Factors That Can Attenuate Vaccinia Virus in Non-Irradiated Bystander Cells

**Figure 7:**
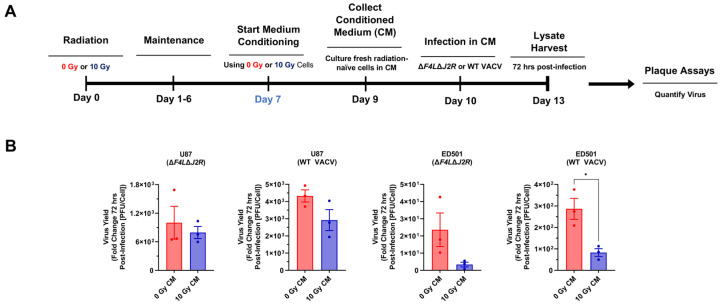
**Irradiated senescence-enriched human glioblastoma cell populations secrete factors that can attenuate vaccinia virus growth.** (**A**) Experimental outline: human U87 and ED501 glioblastoma cell lines were either non-irradiated (0 Gy) or treated with a radiation dose of 10 Gy. Then 7 days later, culture medium was replaced, and conditioned for 48 h. Fresh radiation-naïve glioblastoma cell lines were cultured in this conditioned medium during subsequent virus growth assays. Lysates were harvested immediately after infection (0 h) and 72 h post-infection, then titered in duplicate by plaque assay to assess virus yield. (**B**) Graphs showing fold change in virus yield of oncolytic ∆*F4L*∆*J2R* or wild-type (WT) vaccinia viruses 72 h post-infection in fresh radiation-naïve glioblastoma cells cultured using conditioned medium from either non-irradiated (0 Gy) or 10 Gy treated glioblastoma cells (the cell type infected was matched to the cell type used to condition the media). Cells were infected with 0.03 PFU per cell. **Data information**: data represent three independent experiments, mean ± SEM is shown. Graphs show fold change relative to lysates taken at t = 0. Significance determined by unpaired *t*-test (* = *p* < 0.05).

The SASP is a unique characteristic of senescent cells in which a variety of factors including cytokines, chemokines, mitogens, and proteases are secreted and can cause various downstream effects [67]. The secreted factors from radiation-induced senescent cells generate bystander effects in nearby non-senescent cells [68,69,70]. These reports coupled with the observation that, 7 days following irradiation the senescent cell population in the culture was equally resistant to VACV infection as the proliferating population in the same culture (Figure 3E), led us to hypothesize that radiation exposure may induce the secretion of antiviral factors that might affect virus replication in nearby non-senescent cells. To assess the induction of potential antiviral bystander effects, we performed conditioned medium (CM) experiments as outlined in Figure 7A. Briefly, cellular senescence was induced by irradiating U87 and ED501 cell lines and incubating for 7 days (non-irradiated cells were used as controls), then the cell culture medium was replaced with fresh medium and conditioned for 48 h. Virus growth assays were performed with fresh radiation-naïve GBM cells using CM from irradiated and non-irradiated cells. The growth of oncolytic ∆*F4L*∆*J2R* VACV and WT VACV was assessed 72 h post-infection.

To varying extents, decreases in the virus yield were observed in radiation-naïve U87 and ED501 cells cultured using CM from irradiated senescence-enriched sources compared with cells cultured using CM from non-irradiated controls (Figure 7B). A decreased virus yield was particularly apparent with the ED501 cell line. The mean yield of oncolytic ∆*F4L*∆*J2R* VACV and WT VACV was reduced by ~86% and ~71%, respectively, in radiation-naïve cells cultured using CM from irradiated senescence-enriched sources compared with radiation-naïve cells cultured using CM from non-irradiated controls (Figure 7B), though, only the reduction in WT VACV was significant. With the U87 cell line, the mean yield of oncolytic ∆*F4L*∆*J2R* VACV and WT VACV was reduced by ~21% and ~33%, respectively, in radiation-naïve cells cultured using CM from irradiated senescence-enriched sources compared to radiation-naïve cells cultured using CM from non-irradiated controls (Figure 7B), although reductions in the virus yield were non-significant. These data indicate that irradiated senescence-enriched GBM cell populations may secrete factors that induce bystander cells to impair VACV amplification.

## 4. Discussion

Senescence is a stable growth-arrested cellular state that exhibits unique secretory characteristics. Cellular senescence is not only induced by ionizing radiation, but by a broad range of stimuli including telomere attrition from repeated replicative cycles, upregulated oncogenes, hypoxia, dysfunctional mitochondria, oxidative stress, and genotoxic agents (among others) [71]. Further, senescent cells are implicated in a spectrum of biological processes including developmental activities [72], tissue repair [73,74], inflammation and alerting host immunological systems [75,76,77], age-related pathology [78,79], and cancer [80].

In the context of cancer, the effects of cellular senescence are nuanced, inducing both desirable tumor-suppressive effects [81,82,83] and undesirable tumor progression in a plethora of ways, such as by facilitating cancer relapse [84], enhancing invasiveness [85], contributing to tumor growth [86,87], inducing de novo cancer stem cells [88], and inducing heightened aggressiveness in a subset of cancer cells that escape the senescent state [88,89]. Specific to GBM and radiotherapy, several studies have demonstrated detrimental tumor-promoting effects of GBM cells induced to senesce by ionizing radiation [9,90], and the removal of senescent GBM cells was shown to improve outcomes in preclinical GBM models [91]. Hence, the eradication of radiation-induced senescent cancer cells may optimize therapeutic outcomes, although it is unclear whether or how OV therapy may contribute to the destruction of senescent tumor cells. Despite myriad studies on cellular senescence, reports on virus interactions with senescent cells are limited and the data are conflicting. Some studies indicate that viruses exploit cellular senescence to their benefit [13,14], while others indicate that cellular senescence is associated with antiviral properties [15,16,17,92]. Further, some evidence that OV therapy may offer enhanced killing of senescent cancer cells exists, such as that reported in a study of an oncolytic measles vaccine virus [93]. It is unknown how VACV-based OVs interact with radiation-induced senescent cancer cells. Given that OV therapy is being investigated in combination with radiotherapy (a senescence-inducing agent) to advance the treatment of GBM [4], in conjunction with inconsistent and limited reports regarding VACV interactions with senescent cells, we were interested in better understanding how a VACV-based OV interacts with radiation-induced senescent GBM cells.

We observed that both a VACV-based OV, Δ*F4L*Δ*J2R*, and WT VACV displayed an attenuated phenotype towards irradiated senescence-enriched GBM cell populations. Both viruses exhibited impaired growth kinetics (Figure 2), were less infectious (Figure 3), and showed decreased cytotoxic capabilities (Figure 4) towards irradiated senescence-enriched GBM cell populations compared to non-irradiated GBM cells in vitro. The attenuation of Δ*F4L*Δ*J2R* VACV in irradiated senescence-enriched conditions is not explained by a lack of cellular nucleotide biosynthesis proteins required to compensate the viral gene deletions of *F4L* and *J2R* (Figure 5 and Figure S8). Further, attenuation was also observed with WT VACV, which encodes these genes that are deleted in the mutant. These findings indicate that the senescent state is associated with VACV attenuation and not simply changes in cellular nucleotide biosynthesis levels. Primarily, these data are consistent with the idea that cellular senescence is associated with antiviral activity and highlight an important consideration for optimizing treatment plans that combine VACV-based OVs with radiation. Perhaps treating first with VACV-based OVs—before ionizing radiation induces cellular senescence—may optimize the therapeutic potential of the OV treatment because the OV is able to infect more tumor cells and replicate more effectively, enabling greater amounts of viral progeny to infect/spread through the adjacent tumor cells than there would otherwise be in senescence-enriched conditions. Further investigation of this concept is an obvious next step. Of note, treating GBM mouse models with 10 Gy of radiation followed by Δ*F4L*Δ*J2R* VACV 48 h later produced vastly superior tumor control relative to either modality alone while also inducing immune protection [34]. Therefore, it may also be the case that using OV therapy shortly after radiotherapy (also before radiation induces senescence) avoids senescence-associated antiviral activity.

Our data also indicate that WT VACV is more efficient at producing infectious virions than mutant *ΔF4LΔJ2R* VACV as WT VACV yields were higher throughout virus growth assays (Figure 2 and Figure 7). This observation likely reflects the inherent deficiencies of *ΔF4LΔJ2R* VACV relative to WT VACV due to the deletion of virus-encoded nucleotide biosynthesis machinery. Interestingly, the percentage of VACV-infected cells was similar between WT VACV and *ΔF4LΔJ2R* VACV at 24 h, suggesting that the uptake of the two viruses is similar (Figure 3). It should be emphasized that WT VACV is not suitable as an anti-GBM therapeutic due to toxicities—the LD50 in mice is a mere 10 PFU [94]—whereas *ΔF4LΔJ2R* VACV was safe and effective at a dose of 10,000,000 PFU when used to treat GBM mouse models [34].

Although our studies with VACV/reovirus reinforce the hypothesis that cellular senescence is associated with antiviral activity, this is not an absolute and likely depends on the virus in question. We observed that reovirus, which is a naturally tumor-selective OV, also displayed attenuated growth kinetics in irradiated senescence-enriched U87 cells compared to non-irradiated controls (Figure S6)—corroborating the notion of senescence-associated antiviral activity. In contrast, we did not see attenuation of VSVΔM51-GFP growth in irradiated senescence-enriched GBM cell populations, except marginally, at a low MOI infection with U87 cells (Figure S7)—indicating that radiation-induced senescence had little to no impact on the growth of VSVΔM51-GFP. Of note, the deletion of methionine 51 in the M protein of VSVΔM51-GFP prevents this mutant virus from blocking IFN responses, therefore this virus is attenuated in normal IFN-responsive cells and not in malignant cells (which often have compromised IFN signaling) [41]. Therefore, the fact that VSVΔM51-GFP grew similarly in irradiated and non-irradiated GBM cells is consistent with our data indicating that the type I IFN system is not upregulated in irradiated senescence-enriched GBM cell populations (Figure 6). Further, cellular senescence can promote virus activities. The viral replication of influenza virus and varicella zoster virus was enhanced, respectively, in senescent human bronchial epithelial cells and senescent human dermal cells versus non-senescent cells due to a decreased IFN response in senescent cells relative to non-senescent cells following virus infections [13]. The infectivity of dengue virus was enhanced in senescent human monocytic THP-1 cells relative to non-senescent cells due to increased levels of the viral entry receptor, DC-SIGN, on senescent cells relative to non-senescent cells [14]. Lastly, an oncolytic measles vaccine virus displayed enhanced killing of chemotherapy-induced cancer cells—interestingly, although the mechanism was not elucidated, it was determined to not involve a senescence-associated decrease in IFN responsiveness following virus infection or a senescence-associated upregulation of a virus entry receptor [93]. In conjunction with our data, these studies highlight the nuances of virus interactions with senescent cells and underscore another important consideration for optimizing the combination of OVs with senescence inducers such as radiation: the choice of virus may matter.

Importantly, although VSV was not the focus of our studies, it should be mentioned that some of our data with VSVΔM51-GFP conflict with previous studies investigating WT VSV. Other studies showed that WT VSV was attenuated towards replication-induced, chemotherapy-induced, and oncogene-induced senescent human tumor cells and/or primary mouse cells [17], whereas we observed little to no attenuation of VSVΔM51-GFP in radiation-induced senescence conditions. The contradiction between our study and that conducted by Baz-Martinez et al. (2016) [17], indicates that different senescence-inducing stimuli may induce different types of senescence-associated antiviral activity. The protein contents of the SASP secretome exhibit large variations depending on the stimuli used to induce senescence [95] and factors associated with the SASP have been implicated in partially inducing antiviral properties in exposed cells [17]. It is tempting to speculate that different senescence-inducing stimuli may induce senescence-associated antiviral activity to varying extents due to differing SASP secretomes, depending on the senescence inducer.

We observed an increased expression of NF-κB associated genes but not genes associated with the type I IFN system in irradiated senescence-enriched GBM cell populations compared to non-irradiated controls (Figure 6). NF-κB is a master regulator of the SASP, and SASP factors can reinforce senescence in an autocrine manner while also inducing the senescence of adjacent cells in a paracrine manner [59,96,97]. Given the relevance of NF-κB signaling with senescence, it is not surprising that radiation-induced senescent GBM cells exhibited an increased expression of NF-κB-associated genes. Further, the activation of NF-κB is a well-established antiviral signaling system [57] and VACV encodes a plethora of viral inhibitors to thwart host NF-κB activation in order to operate optimally [98,99,100,101]. Given the role NF-κB plays in antiviral signaling, our findings suggest that the activation of NF-κB pathways may have contributed to the decreased susceptibility of radiation-induced senescent GB cells to VACV.

Lastly, CM experiments revealed the suppression of Δ*F4L*Δ*J2R* VACV and WT VACV growth in non-irradiated GBM cells cultured in media taken from irradiated senescence-enriched GBM cell populations versus media from non-irradiated controls (Figure 7). This finding indicates that factors secreted by irradiated senescence-enriched GBM cell populations induce an antiviral bystander effect in non-irradiated GBM cells. At least one other study using WT VSV corroborates the notion of a senescence-associated antiviral bystander effect. The cytotoxicity of WT VSV towards A549 lung cancer cells and primary mouse embryo fibroblast cells was impaired when cultured using CM from senescent versus non-senescent cells [17]. Our data also suggest that the SASP is implicated in bystander paracrine antiviral activation. Further investigation should include cytokine arrays to identify the specific SASP components that may be involved in this senescence-associated antiviral activity.

## 5. Conclusions

In conclusion, we show that both oncolytic Δ*F4L*Δ*J2R* VACV and WT VACV display less productive infectivity, attenuated growth, and decreased cytotoxic capabilities in irradiated senescence-enriched GBM cell populations. The resistance of radiation-induced senescent GBM cells to VACV may be in part mediated by NF-κB signaling and SASP-associated bystander effects. This research implicates radiation-induced cellular senescence as an antiviral state that impairs VACV. Further, our findings underscore important treatment planning considerations for the combination of VACV-based oncolytic therapies with senescence-inducing agents such as radiotherapy.

## Data Availability

All data supporting the findings of this study are available within the article, Supplementary Material files, and from the corresponding author upon reasonable request.

## Supplementary Materials

The following supporting information can be downloaded at: https://www.mdpi.com/article/10.3390/cancers15133341/s1, Figure S1: Representative images of non-irradiated and irradiated CellTrace™ Violet stained U87 human glioblastoma cells (also treated with wild-type vaccinia virus in this case); Figure S2: Verification of radiation-induced senescence in irradiated glioblastoma cells; Figure S3: ΔCt values used to calculate significant differences of SASP gene expression between non-irradiated and irradiated human glioblastoma cell lines; Figure S4: Growth attenuation of vaccinia virus occurs in additional irradiated senescence-enriched human glioblastoma cell lines; Figure S5: Growth of vaccinia virus is attenuated in irradiated senescence-enriched U87 human glioblastoma cells cultured in normal-serum or low-serum conditions; Figure S6: Reovirus growth is absent or attenuated in irradiated senescence-enriched human glioblastoma cell lines; Figure S7: Oncolytic vesicular stomatitis virus growth in irradiated senescence-enriched human glioblastoma cell lines and non-irradiated controls; Figure S8: J2R- and F4L-deleted vaccinia viruses display attenuated growth in irradiated senescence-enriched human glioblastoma cell lines; Figure S9: ΔCt values used to calculate significant differences of IFN- and NF-κB-associated gene expression between non-irradiated and irradiated human glioblastoma cell lines; File S1: Uncropped immunoblots. Table S1. Primers used in RT-qPCR studies.
