# Supplementary material for: Radiation-Induced Cellular Senescence Reduces Susceptibility of Glioblastoma Cells to Oncolytic Vaccinia Virus
Source: Cancers (Basel). 2023 Jun 25;15(13):3341. doi: 10.3390/cancers15133341 (PMC10341205; doi:10.3390/cancers15133341)
Supplement: Supplementary file 1 [file cancers-15-03341-s001.zip › Supplementary Figures (with Table S1).pdf]

Table S1. Primers used in RT-qPCR studies.

Target Gene (human)	Forward Primer	Reverse Primer
GROα	5’- GAA AGC TTG CCT CAA TCC TG -3’	5’- CAC CAG TGA GCT TCC TCC TC -3’
GROβ	5’- AAC TGC GCT GCC AGT GCT -3’	5’- CCC ATT CTT GAG TGT GGC TA -3’
GM-CSF	5’- GGC CCC TTG ACC ATG ATG -3’	5’- TCT GGG TTG CAC AGG AAG TTT -3’
IL-6	5’- CCG GGA ACG AAA GAG AAG CT -3’	5’- GCG CTT GTG GAG AAG GAG TT -3’
IL-8	5’- CTT TCC ACC CCA AAT TTA TCA AAG -3’	5’- CAG ACA GAG CTC TCT TCC ATC AGA -3’
IFNβ	5’- AGG ACA GGA TGA ACT TTG AC -3’	5’- TGA TAG ACA TTA GCC AGG AG -3’
MX1	5’- TTC AGC ACC TGA TGG CCT A -3’	5’- AAA GGG ATG TGG CTG GAG AT -3’
ISG15	5’- GCG AAC TCA TCT TTG CCA GTA -3’	5’- CCA GCA TCT TCA CCG TCA G -3’
IκBα	5’- GCT GAA GAA GGA GCG GCT ACT -3’	5’- TCG TAC TCC TCG TCT TTC ATG GA -3’
IL1β	5’- CCC AAC TGG TAC ATC AGC AC -3’	5’- GGA AGA CAC AAA TTG CAT GG -3’
TNFα	5’- CCC GAG TGA CAA GCC TGT AG -3’	5’- GAT GGC AGA GAG GAG GTT GAC -3’
18S rRNA	5’- CCC TAT CAA CTT TCG ATG GTA GTC G -3’	5’- CCA ATG GAT CCT CGT TAA AGG ATT T -3’

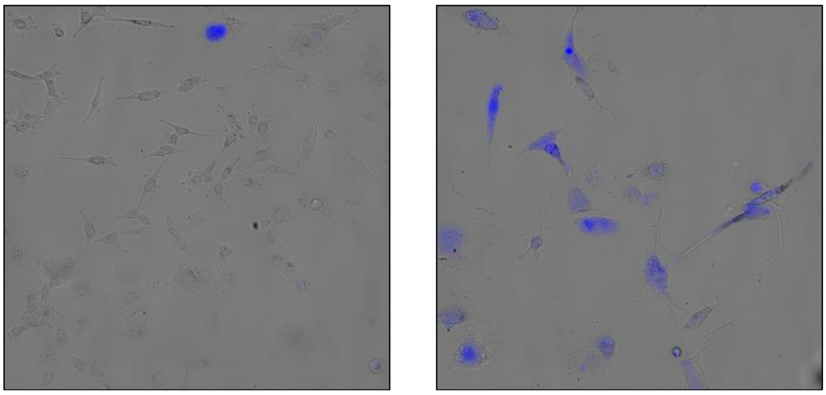

Figure S1. Representative images of non-irradiated and irradiated CellTrace™ Violet stained U87 human glioblastoma cells (also treated with wild-type vaccinia virus in this case). Human U87 glioblastoma cells were stained with a fluorescent cell proliferation marker (CellTrace™ Violet; CTV) then were either non-irradiated (0 Gy) or treated with a radiation dose of 10 Gy. 7 days later, cells were infected with 3.0 PFU per cell of wild-type vaccinia virus. 24 hours post-infection, cells were fixed then immunostained with an antibody against the late A27 VACV protein and imaged the next day using fluorescence microscopy. Blue cells in the images were scored as high CTV intensity (CTV^{h}). Grey cells in the images were scored as low CTV intensity (CTV^{lo}).

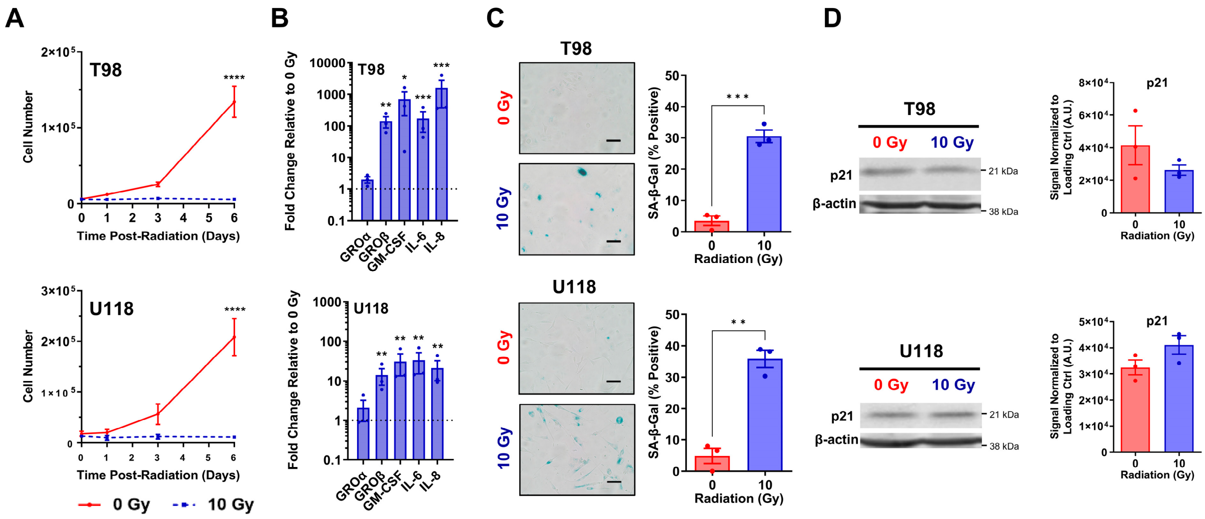

Figure S2. Verification of radiation-induced senescence in irradiated glioblastoma cells. Human T98 and U118 glioblastoma cell lines were either non-irradiated (0 Gy) or treated with a radiation dose of 10 Gy then evaluated for markers of cellular senescence. **(A)** Cellular growth curves showing the change in cell number over time following the indicated radiation treatments. Trypan blue assays were performed to quantify total cells at the indicated time points. **(B)** Fold change in expression of senescence-associated secretory phenotype (SASP) genes in 10 Gy treated cells relative to 0 Gy treated cells 7 days following radiation treatments, based on RT-qPCR analysis. 18S rRNA levels were used for normalization. **(C)** Representative images of cells assessed for senescence-associated-β-galactosidase activity (left panels) and quantification of cells positive for senescence-associated-β-galactosidase activity (right panels) 7 days following the indicated radiation treatments. **(D)** Representative immunoblots showing p21 protein levels (left panels) and quantification of band signals (arbitrary units; A.U.) normalized to β-tubulin loading controls (right panels) 7 days following radiation treatments. **Data information:** Data represent 3 independent experiments, mean ± SEM is shown. For (A), significance was determined by two-way ANOVA. For (B), asterisks indicate significance from unpaired t-test comparing ΔCt values (ΔCt values shown in **Supplementary Figure S3**). For (C), unpaired t-test was used to determine significance and scale bar = 100 μm. (* = $p < 0.05$; ** = $p < 0.01$; *** = $p < 0.001$; **** = $p < 0.0001$). For (D), the uncropped immunoblots are shown in **Supplementary Material File S1**.

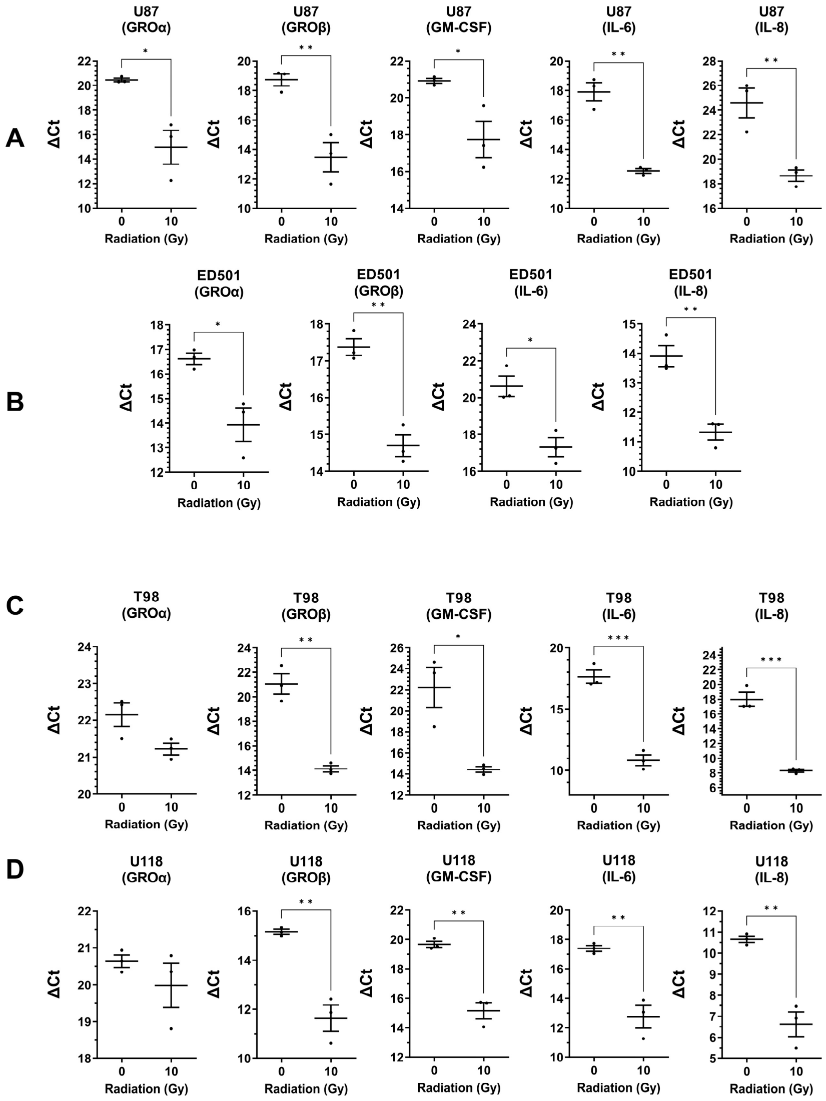

Figure S3. ΔCt values used to calculate significant differences of SASP gene expression between non-irradiated and irradiated human glioblastoma cell lines. ΔCt values of the indicated senescence-associated secretory phenotype (SASP) genes based on RT-qPCR analysis of 0 Gy (non-irradiated) or 10 Gy treated human glioblastoma cell lines 7 days post-irradiation. 18S rRNA levels were used for normalization. The glioblastoma cell lines are indicated: (A) U87, (B) ED501, (C) T98, and (D) U118. Data information: Data represent 3 independent experiments, mean ± SEM is shown. ΔCt values calculated by subtracting the Ct value of the 18S gene from the Ct value of the target gene within the same treatment condition. Significance determined using unpaired t-test (* = $p < 0.05$; ** = $p < 0.01$; *** = $p < 0.001$).

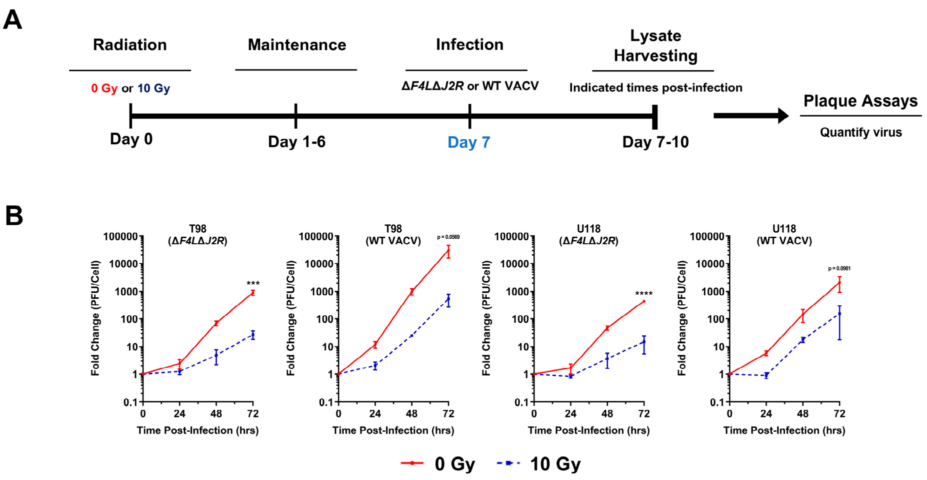

Figure S4. Growth attenuation of vaccinia virus occurs in additional irradiated senescence-enriched human glioblastoma cell lines. (A) Experimental outline. Human T98 and U118 glioblastoma cell lines were either non-irradiated (0 Gy) or treated with a radiation dose of 10 Gy. 7 days later, cells were infected with 0.03 PFU per cell of the indicated vaccinia viruses. Lysates were harvested at the indicated times and titered by plaque assay to assess virus yield. (B) Growth curves showing amplification of oncolytic Δ*F4L*Δ*J2R* and wild-type (WT) vaccinia viruses in 0 Gy (non-irradiated; solid red lines) and 10 Gy (dashed blue lines) treated human T98 and U118 glioblastoma cell lines. Data information: Data represent 3 independent lysates titered in duplicate. Mean ± SEM is shown. Graphs show fold change relative to lysates taken at t = 0. Significance determined by two-way ANOVA analysis (*** = p < 0.001; **** = p < 0.0001).

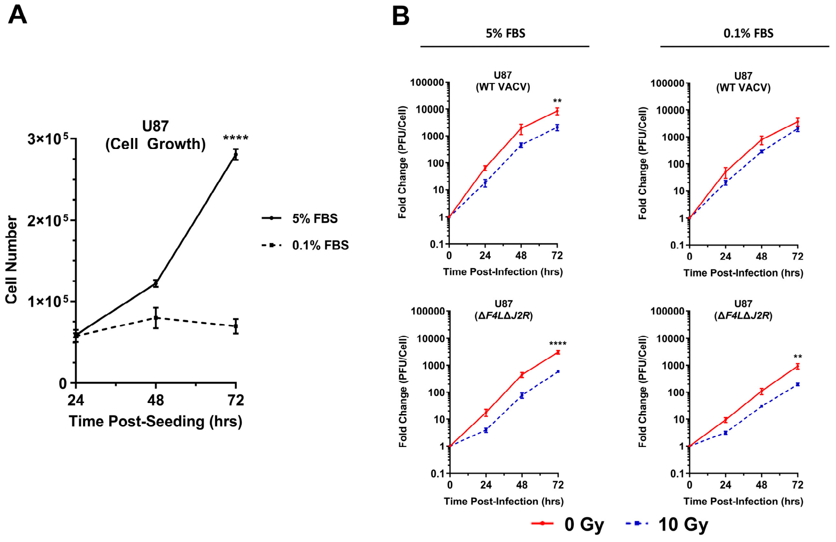

Figure S5. Growth of vaccinia virus is attenuated in irradiated senescence-enriched U87 human glioblastoma cells cultured in normal-serum or low-serum conditions. (A) Cellular growth curves showing the change in cell number over time of human U87 glioblastoma cells cultured in normal-serum (5% FBS; solid line) or low-serum (0.1% FBS; dashed line) conditions. Trypan blue assays were performed to quantify total cells at the indicated time points. (B) Growth curves showing amplification of oncolytic Δ*F4L*Δ*J2R* and wild-type (WT) vaccinia viruses in non-irradiated (0 Gy; red solid lines) and 10 Gy (dashed blue lines) treated human U87 glioblastoma cells. Cells were infected 7 days after radiation treatments with 0.03 PFU per cell and cultured in normal-serum (5% FBS; left panels) or low-serum (0.1% FBS; right panels) conditions. Lysates were harvested at the indicated times and titered in duplicate by plaque assay to assess virus yield. Data information: Data represent 3 independent experiments. Mean ± SEM is shown. For (B), graphs show fold change relative to lysates taken at t = 0. Significance determined by two-way ANOVA analysis (** = p < 0.01; **** = p < 0.0001).

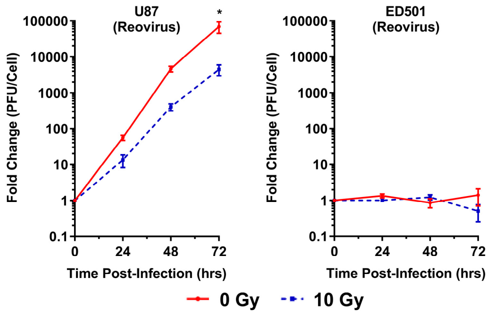

Figure S6. Reovirus growth is absent or attenuated in irradiated senescence-enriched human glioblastoma cell lines. Growth curves showing amplification of reovirus (T3Dearing) in non-irradiated (0 Gy; solid red lines) and 10 Gy (dashed blue lines) treated human U87 and ED501 glioblastoma cell lines. 7 days following radiation treatments, cells were infected with reovirus (0.03 PFU per cell). Lysates were harvested at the indicated times and titered by plaque assay to assess virus yield. **Data information:** Data represent 3 independent lysates titered in duplicate. Mean ± SEM is shown. Graphs show fold change relative to lysates taken at t = 0. Significance determined by two-way ANOVA analysis (* = $p < 0.05$).

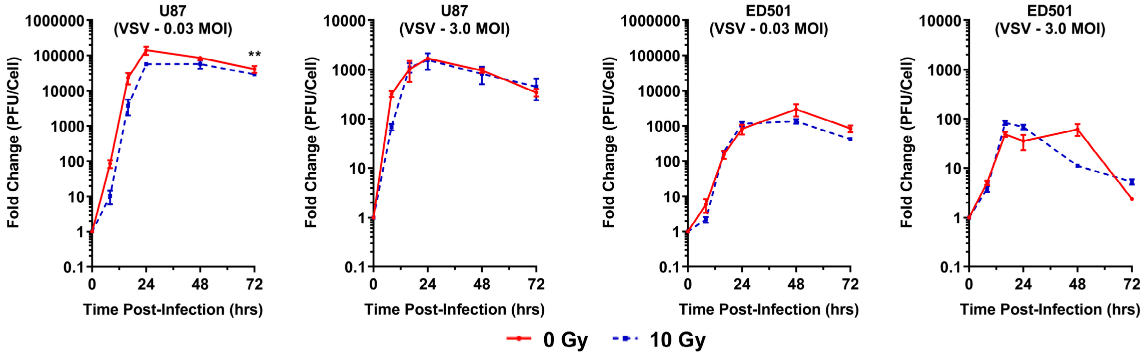

Figure S7. Oncolytic vesicular stomatitis virus growth in irradiated senescence-enriched human glioblastoma cell lines and non-irradiated controls. Growth curves showing amplification of VSVΔM51-GFP in non-irradiated (0 Gy; solid red lines) and 10 Gy (dashed blue lines) treated human U87 and ED501 glioblastoma cell lines. 7 days following radiation treatments, cells were infected with VSVΔM51-GFP (0.03 PFU per cell or 3.0 PFU per cell). Lysates were harvested at the indicated times and titered by plaque assay to assess virus yield. Data information: Data represent 3 independent lysates titered in duplicate. Mean ± SEM is shown. Graphs show fold change relative to lysates taken at t = 0. Significance determined by two-way ANOVA analysis (** = $p < 0.01$).

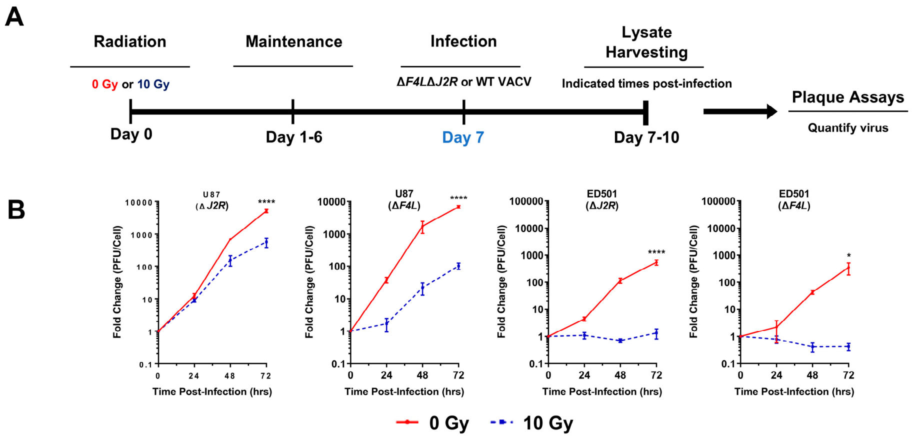

Figure S8. *J2R*- and *F4L*-deleted vaccinia viruses display attenuated growth in irradiated senescence-enriched human glioblastoma cell lines. (A) Human U87 and ED501 glioblastoma cell lines were either non-irradiated (0 Gy) or treated with a radiation dose of 10 Gy. 7 days later, cells were infected with 0.03 PFU per cell of the indicated vaccinia viruses. Lysates were harvested at the indicated times and titered by plaque assay to assess virus yield. (B) Growth curves showing amplification of *J2R*-deleted and *F4L*-deleted vaccinia viruses in non-irradiated (0 Gy; solid red lines) and 10 Gy (dashed blue lines) treated human U87 and ED501 glioblastoma cell lines. Data information: Data represent 3 independent lysates titered in duplicate. Mean ± SEM is shown. Graphs show fold change relative to lysates taken at t = 0. Significance determined by two-way ANOVA analysis (* = $p < 0.05$; **** = $p < 0.0001$).

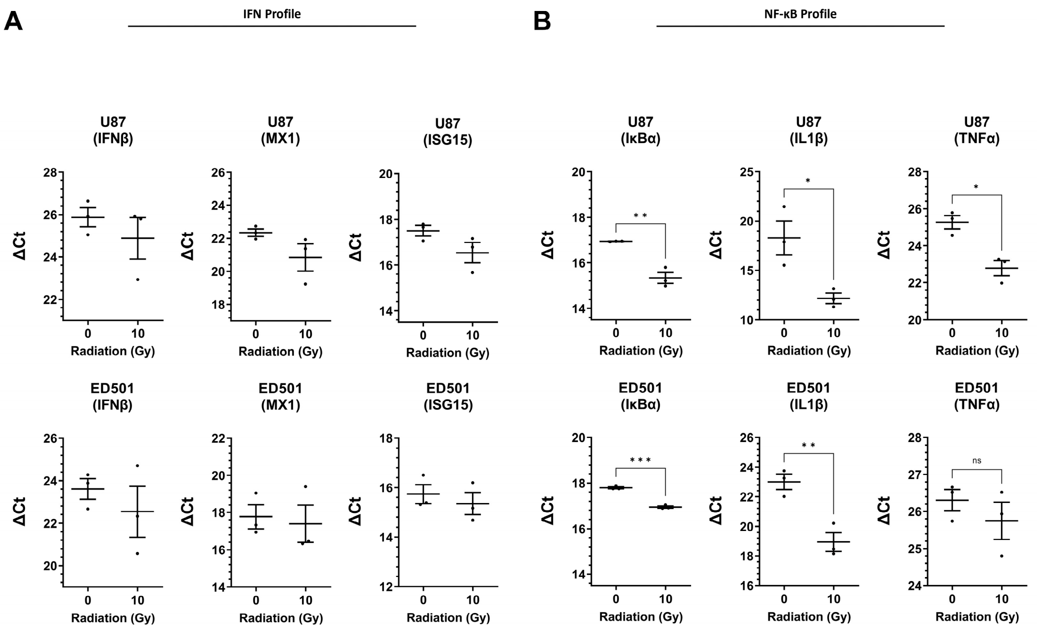

Figure S9. ΔCt values used to calculate significant differences of IFN- and NF-κB-associated gene expression between non-irradiated and irradiated human glioblastoma cell lines. ΔCt values of: (A) type I interferon (IFN) related genes, (B) NF-κB-associated genes; based on RT-qPCR analysis of non-irradiated (0 Gy) and 10 Gy treated human U87 and ED501 glioblastoma cell lines 7 days following radiation treatments. 18S rRNA levels were used for normalization. Data information: Data represent 3 independent experiments, mean ± SEM is shown. ΔCt values calculated by subtracting the Ct value of the 18S gene from the Ct value of the target gene within the same treatment condition. Significance determined using unpaired t-test (* = $p < 0.05$; ** = $p < 0.01$; *** = $p < 0.001$).
