# Supplementary material for: Radiation-Induced Cellular Senescence Reduces Susceptibility of Glioblastoma Cells to Oncolytic Vaccinia Virus
Source: Cancers (Basel). 2023 Jun 25;15(13):3341. doi: 10.3390/cancers15133341 (PMC10341205; doi:10.3390/cancers15133341)
Supplement: Supplementary file 1 [file cancers-15-03341-s001.zip › Supplementary Material File S1 (Uncropped Immunoblots).pdf]

U87 Blots

Lanes:
1 – Trial 1: U87 (0 Gy)
2 – Trial 1: U87 (10 Gy)
3 – Trial 2: U87 (0 Gy)
4 – Trial 2: U87 (10 Gy)
5 – Trial 3: U87 (0 Gy)
6 – Trial 3: U87 (10 Gy)

Targets

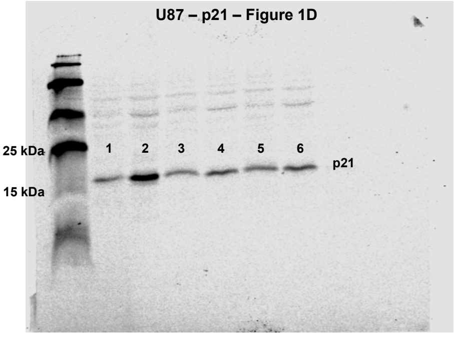

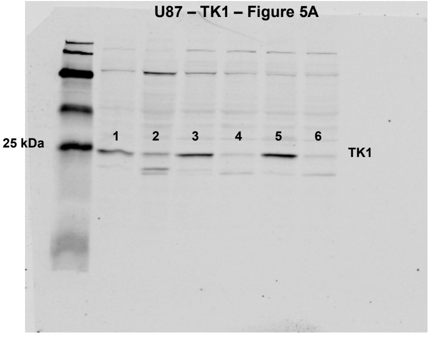

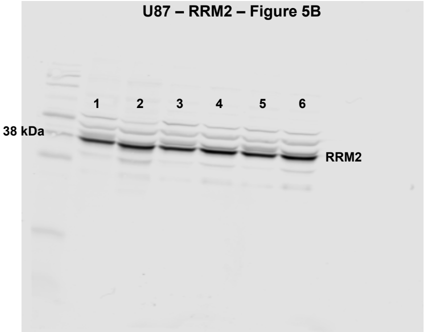

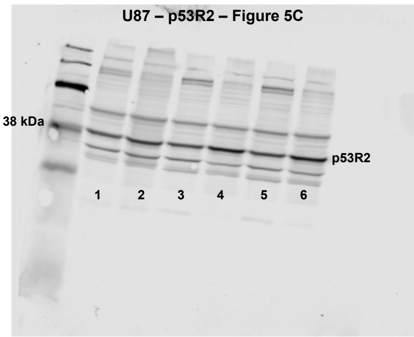

Loading Controls

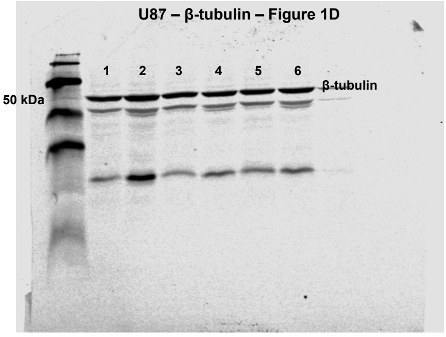

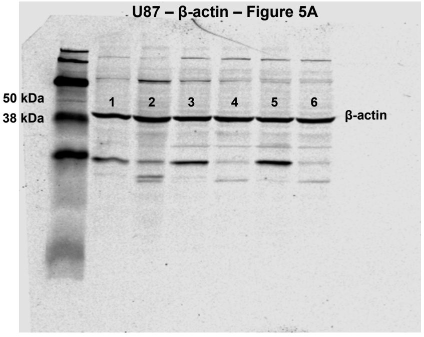

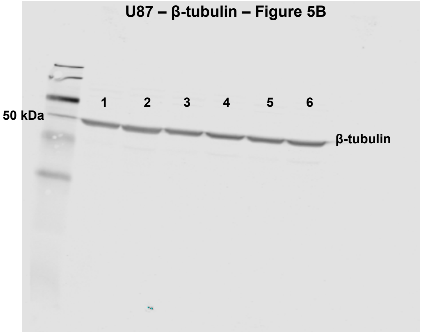

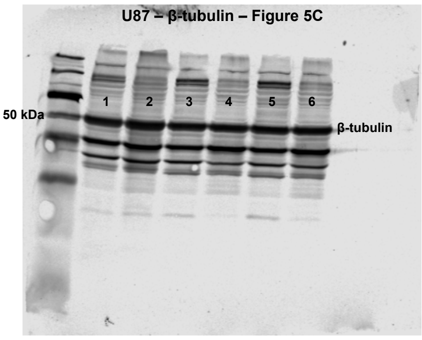

ED501 Blots

Targets

Loading Controls

Lanes:

1 – Trial 1: ED501 (0 Gy)
2 – Trial 1: ED501 (10 Gy)
3 – Trial 2: ED501 (0 Gy)
4 – Trial 2: ED501 (10 Gy)
5 – Trial 3: ED501 (0 Gy)
6 – Trial 3: ED501 (10 Gy)

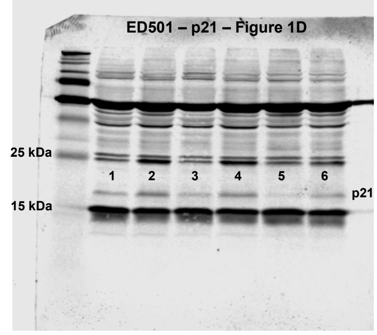

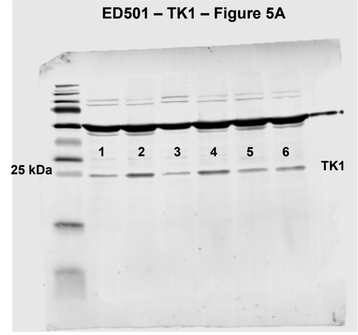

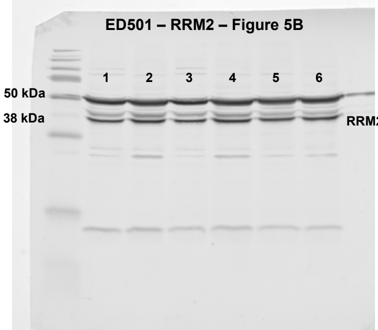

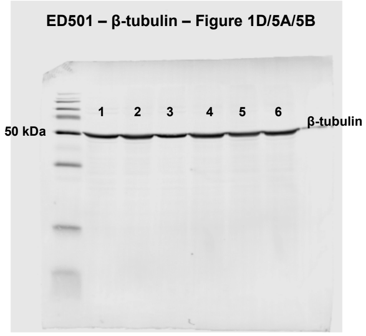

Same blot probed with p21, TK1, RRM2

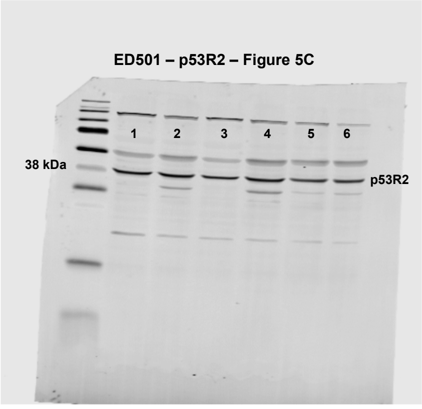

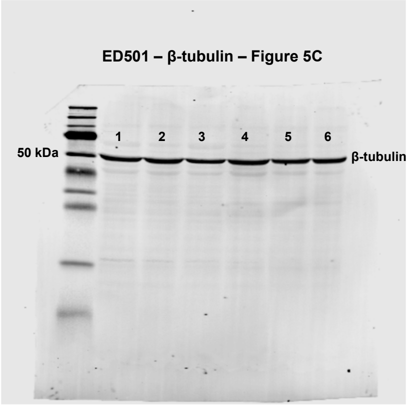

T98 Blots

Targets

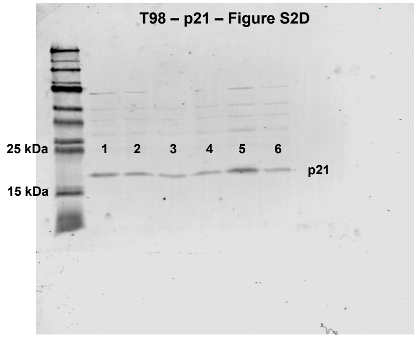

Loading Controls

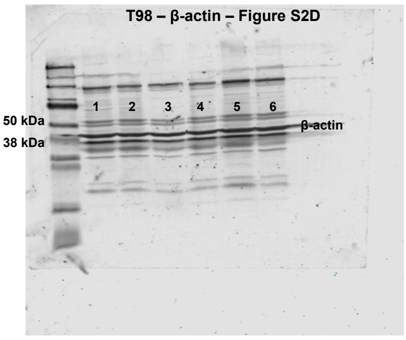

Lanes:

1 – Trial 1: T98 (0 Gy)

2 – Trial 1: T98 (10 Gy)

3 – Trial 2: T98 (0 Gy)

4 – Trial 2: T98 (10 Gy)

5 – Trial 3: T98 (0 Gy)

6 – Trial 3: T98 (10 Gy)

U118 Blots

Targets

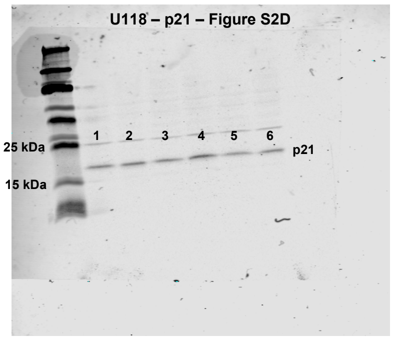

Loading Controls

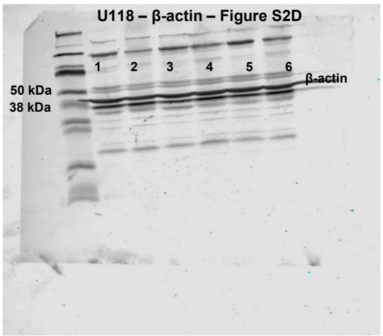

Lanes:

1 – Trial 1: U118 (0 Gy)

2 – Trial 1: U118 (10 Gy)

3 – Trial 2: U118 (0 Gy)

4 – Trial 2: U118 (10 Gy)

5 – Trial 3: U118 (0 Gy)

6 – Trial 3: U118 (10 Gy)
